# Association of Working Hours and Patient Safety Competencies with Adverse Nurse Outcomes: A Cross-Sectional Study

**DOI:** 10.3390/ijerph16214083

**Published:** 2019-10-24

**Authors:** Youn-Jung Son, Eun Kyoung Lee, Yukyung Ko

**Affiliations:** 1 Red Cross College of Nursing, Chung-Ang University, 84 Heukseok-ro, Heukseok-dong, Dongjak-Gu, Seoul 06974, Korea; yjson@cau.ac.kr; 2 Nursing Department, Soonchunhayng University Hospital Cheonan, 31 Suncheonhyang 6-Gil, Dongnam-gu, Cheonan-si, Chingcheongnam-do, Cheonan 31151, Korea; Ekrhee76@gmail.com; 3 Department of Nursing, College of Medicine, Wonkwang University, 460 Iksandae-ro, Iksan, Jeonbuk 54538, Korea

**Keywords:** adverse outcome, nursing, patient safety, professional competence

## Abstract

The environment of health organizations can determine healthcare quality and patient safety. Longer working hours can be associated with nurses’ health status and care quality, as well as work-related hazards. However, little is known about the association of hospital nurses’ working hours and patient safety competencies with adverse nurse outcomes. In this cross-sectional descriptive study, convenience sampling was employed to recruit 380 nurses from three tertiary care hospitals in South Korea. Data were collected using structured questionnaires from May to June 2016. Hierarchical linear regression analysis was used to identify the association of working hours and patient competencies with adverse nurse outcomes among 364 participants selected for analysis. Most nurses worked over 40 h/week. Working hours (*β* = 0.202, *p* < 0.001) had the strongest association with adverse nurse outcomes. Low perceived patient safety competencies (*β* = −0.179, *p* = 0.001) and frequently reporting patient safety accidents (*β* = 0.146, *p* = 0.018) were also correlated with adverse nurse outcomes. Nursing leaders should encourage work cultures where working overtime is discouraged and patient safety competencies are prioritized. Further, healthcare managers must formulate policies that secure nurses’ rights. The potential association of overtime with nurse and patient outcomes needs further exploration.

## 1. Introduction

Patients’ health problems and their care needs are constantly increasing with population aging [1,2,3]. Thus, healthcare environments have gradually become more specialized and demanding [4]. Within these changing healthcare systems, the requirement of high-quality and optimal patient care from nurses has remained constant [5]. South Korea had 5.6 practicing nurses, including nursing aides, per 1000 people in 2014, far lower than the Organization for Economic Co-operation and Development average of 9.6 [6]. Moreover, in South Korea, turnover among nurses with less than one year of experience is a matter of particular concern [7]. In response to this shortage, the government has increased the number of nursing schools to expand the supply of nurses. These efforts have increased the number of licensed nurses, but unfortunately failed to increase the number of practicing nurses [8]. In this context, a previous study reported that only 28.8% of South Korean hospitals were classified as having a better than average work environment compared to up to 45.5% of hospitals in other countries [9]. This implies that poor work environments make it difficult for nurses to perform their roles. 

Work environments have been broadly discussed as a critical issue in attracting and retaining experienced and professional nurses in medical settings [10,11]. The environment of health organizations can determine the quality and safety of healthcare. The characteristics of work environments that have been identified as obstacles to good nursing care include concerns about workplace stress, insufficient staff, and time demands [12]. From the perspective of ensuring patient safety (PS), there is increasing concern worldwide about the impact of nurses’ long working hours, despite the growing capabilities of healthcare systems [13,14]. Extended working hours can lead to short rest periods between shifts, which can not only affect nurses’ health status but also increase their risk for burnout and work-related hazards [15]. Working overtime excessively was found to be associated with occurrences of medication errors, pressure ulcers, injuries from falls, catheter-associated urinary tract infection, and hospital-acquired infections [11]. Moreover, it has been demonstrated that among nurses, working overtime is associated with inadequate sleep quality and fatigue [16], and consequently, the occurrence of needle stick injury [17]. Trinkoff, Le, Geiger-Brown, Lipscomb, and Lang [18] found that a quarter of their study sample worked for more than 40 h/week and 9% of registered nurses worked for more than 60 h/week. The policies associated with working hours have a crucial effect on patient care and outcomes. In South Korea, about 56.7% of physicians and 82.1% of nurses work in hospitals that are open 24 h a day, 365 days a year (as of 2011). According to the Labor Standards Act, the number of working hours in a week cannot exceed 40, excluding breaks [19]. Despite this, staff nurses in hospitals report working an average of 46.6 h per week [20]. In particular, working overtime and extended working hours are common among nurses in university hospitals [21]. However, data regarding the statistics on working hours and overtime among hospital nurses and their association with nursing outcome are limited in South Korea.

PS is increasingly recognized as an important issue in care quality [5]. Therefore, educational institutions or the healthcare delivery system, which collaborate with the controlling institution, have enforced strategic mandates for the effective provision of PS education to nurses [22,23]. PS competency is defined as knowledge, attitudes, and skills concerning PS, which are needed for the safe provision of healthcare services [23]. The Canadian PS Institute has made recommendations for six PS competencies: “contributing to PS culture”, “working in teams for PS”, “communicating effectively for PS”, “managing safety risks”, “making the best of human and environmental factors”, and “identifying, answering, and uncovering adverse events” [22]. Frontline healthcare workers’ PS competencies play a vital role in providing high-quality care with desirable outcomes.

Adverse nurse outcomes include physical, interpersonal, and emotional consequences in the workplace, such as physical exhaustion; skipping breaks; feeling overwhelmingly responsible for more than one patient; insufficient time for documentation; verbal abuse and violence from a patient, family, or doctor; and concerns about poor care [24]. As nurses are frontline care providers, organizations or supervisors often burden them with reducing the risk of PS incidents [25]. This burden is associated with increased turnover and job stress [24] and lower organizational commitment [25]. There are bidirectional associations between nurse and patient outcomes. For instance, nurses feel dissatisfied with their work conditions when they are unable to provide efficient care [26], which can, in turn, increase patient complaints related to healthcare service quality. Further, poor nurse outcomes can lead to higher costs because of low productivity, workers’ compensation claims, and high turnover.

Until now, most studies regarding PS issues have focused on negative patient outcomes such as nosocomial infections, falls, rehospitalizations, and mortality. Despite the importance of understanding the relationship between working hours, PS competencies, and adverse nurse outcomes, few studies have conducted such direct examinations. PS at medical institutes is a matter that needs continued quality improvement [5]. For this reason, previous studies have emphasized the importance of healthy nursing work environments and establishing a culture of PS [5,8]. Considering the need to solve the problem of the nursing shortage in South Korea, the present study aimed to examine the correlation of working hours and PS competencies with adverse nurse outcomes, with the ultimate goal of determining ways to improve nursing outcomes. 

The detailed objectives were as follows: first, to identify the differences in PS competencies and adverse nurse outcomes in different working hour groups; second, to identify the characteristics of the participants in different working hour groups; and, finally, to explore the association of working hours and PS competencies with adverse nurse outcomes as an indicator of organizational performance in South Korean nurses. 

## 2. Materials and Methods

### 2.1. Study Design and Participants

A cross-sectional descriptive design was adopted to conduct a survey examining registered nurses of all levels in the acute care hospital setting. Nurses working at three tertiary care hospitals affiliated to Soonchunhyang University in South Korea were recruited through convenience sampling. Each of the three study sites, located in Cheonan, Bucheon, and Seoul, had similar hospital and organizational performance, including types of services provided, number of beds (750–800), patient types, average hospital stay, ownership, and payment systems, as well as regulations regarding nurse staffing.

The inclusion criteria were as follows: nurses who had at least one year of experience working at one of the three tertiary care hospitals and registered nurses working three eight-hours shifts a day at inpatient units such as medical-surgical units, intensive care units, emergency rooms, and hemodialysis rooms. Nurses from outpatient clinics, operating rooms, and recovery rooms were excluded because their work differs from that of nurses from inpatient units. In addition, nurses who were on leave during the entire data collection period were excluded from the study. 

Power calculations were conducted with G*Power 3.1.5. [27]. The sample size required for the hierarchical regression analysis with an R^2^ increase was calculated according to the following parameters: number of predictors = 10, power = 99%, and medium effect size = 0.10. Consequently, 380 nurses were enrolled; however, 16 questionnaires were excluded owing to incomplete answers.

Consequently, the data of 138 nurses from the hospital in Cheonan, 114 nurses from the hospital in Bucheon, and 112 nurses from the hospital in Seoul were analyzed. The participants (*N* = 364) were women with a mean age of 30.02 ± 6.24 years (range = 23–51 years). 

### 2.2. Measurements

#### 2.2.1. Sociodemographic and Work-Related Characteristics

The sociodemographic and work-related aspect of the structured questionnaire included 10 items on gender, age, marital status, education, work units, current position, career length, PS education, frequency of reporting PS accidents, and working hours. 

With regard to working hours, this study was based on the 4th Korean Working Conditions Survey (KWCS), conducted in 2014 [28]. The KWCS identifies current trends in working conditions of South Korean employees. Weekly working hours were determined through the following question: “How many hours do you usually work per week?” Lunch break and commuting time were excluded from working hour calculations. Based on the KWCS criteria, working hours in this study were categorized into three groups: <40 h/week, 40–49 h/week, and ≥50 h/week. 

#### 2.2.2. PS Competencies

To measure nurses’ perceptions of their PS competencies, we used the “Health Professional Education in PS Survey”, developed by Ginsburg, Castel, Tregunno, and Norton [29]. This is a validated 16-item questionnaire assessing self-reported PS competencies that includes six dimensions: working in teams with other healthcare professionals (three items), efficient communication (three items), management of safety risks (three items), understanding human and environmental factors (two items), recognition of and response to adverse events (two items), and safety culture (three items). Cronbach’s alpha was 0.83 in the original study and 0.91 in this study.

#### 2.2.3. Adverse Nurse Outcomes 

Adverse nurse outcomes were measured using a questionnaire developed by Al-Kandari and Thomas [24], which comprised 15 items including physical and emotional exhaustion, skipping breaks, feeling responsible for more than one patient, inadequate help and time, facing verbal and physical abuse, worries about the quality of care, complaints from patients or families, and being injured at work. The 15 items in the final questionnaire were translated into Korean through a forward-backward translation process performed by two bilingual native speakers. 

To improve content validity, we received expert opinions regarding the translated Korean version from two nursing professors and five nursing managers. To calculate the content validity index (CVI), the seven experts rated each item on a five-point Likert scale. Items with CVI scores greater than 0.79 were considered appropriate [30]; as the scale’s CVI was 0.89, none of the items needed modification.

Adverse nurse outcomes experienced over the past year were measured on a four-point Likert scale (4: “*frequently experienced*” to 1: “*not experienced at all*”). Higher scores indicated more job pressure. In this study, Cronbach’s alpha was 0.84.

### 2.3. Data Collection

Data were obtained through structured questionnaires from May to June 2016. The questionnaires were distributed by the nurse managers, and the participants returned the forms in a sealed box or return envelope. Participants received the questionnaire with an information sheet describing the voluntary nature of participation, the right to withdraw at any time, and the purpose of the study with measures to preserve confidentiality. 

### 2.4. Data Analysis

Continuous variables were presented as means and standard deviations. For discrete variables, groups were compared using *χ*^2^ tests. One-way analysis of variance was used to examine the differences in the means of PS competencies and adverse nurse outcomes as per participants’ working hours. Dummy variables were created for categorical variables. A hierarchical linear regression analysis was conducted to investigate the association of working hours and perceived PS competencies with adverse nurse outcomes.

Multicollinearity was detected using the variation inflation factor (VIF) and tolerance. The tolerance values for the models ranged from 0.31 to 0.59, and the VIF values varied from 1.01 to 3.45. A tolerance of ≤0.1 and a VIF of ≥10 may indicate collinearity [31]. In our study, there was no multicollinearity among variables. Namely, the VIF value of <10 and tolerance value of >0.2 were acceptable. Cook’s distance was assessed to detect any outliers influencing the regression models. A two-tailed *p*-value of ≤0.05 was considered statistically significant. All analyses were performed using SPSS 23.0 (SPSS Inc., Chicago, IL, USA).

## 3. Results

### 3.1. Relationship between Participant Characteristics and Working Hours

Participants’ mean age was 29.84 ± 5.42 years. Most were married (*n* = 240, 65.9%), and 202 participants (55.5%) had a bachelor’s degree. The mean total working hours/week were 44.6 ± 6.16 (range = 34.0–58.0). When participants were divided into three groups according to working hours, there were significant differences by work unit. While only 14 participants in the intensive care unit (15.7%) reported working ≥50 h, among nurses working in internal medicine wards, surgery wards, emergency departments, and hemodialysis units, the number of those working <40 h was lower than those who reported working 40–49 h or ≥ 50 h (χ^2^ = 23.65, *p* = 0.003). There were no significant differences between the working hour groups regarding age, marital status, educational level, job position, clinical career, PS education, or accident reporting in the past year (Table 1).

### 3.2. PS Competencies and Degree of Adverse Nurse Outcomes Per Working Hour Group

The three working hour groups were compared in terms of their perceived PS competencies and adverse nurse outcomes (Table 2). The <40 h group had the highest perceived PS competencies (56.00 ± 6.92), differing significantly from the 40–49 and ≥50 h groups. The ≥50 h group had the highest adverse nurse outcomes score (48.53 ± 5.58), differing significantly from the <40 and 40–49 h groups.

### 3.3. Factors Associated with Adverse Nurse Outcomes

Table 3 shows the results of the hierarchical regression analysis with age, marital status, educational level, working unit, job position, clinical career, PS education, and accident reporting frequency in the past year entered in step 1 and PS competencies entered in step 2. In step 1, having reported more than three accidents in the past year (*β* = 0.149, *p* = 0.018) and working hours exceeding 40 h/week (*β* = 0.223, *p* < 0.001) were correlated with adverse nurse outcomes. With PS competencies entered in step 2, having reported more than three accidents in the past year (*β* = 0.146, *p* = 0.018), working hours exceeding 40/week (*β* = 0.202, *p* < 0.001), and higher PS competencies (*β* = −0.179, *p* = 0.001) were correlated with adverse nurse outcomes.

## 4. Discussion

Positive nursing performance and outcomes are highly important as they are directly linked to PS [32]. We investigated nurses’ working hours, PS competencies, and adverse nurse outcomes and aimed to identify the factors correlated with adverse nurse outcomes, ultimately seeking to determine ways to improve PS and boost the quality of nurses’ work. We found that long working hours were strongly associated with adverse nurse outcomes.

First, we examined nurses’ general characteristics related to their working hours. We found that working hours significantly differed across work units. Ko and Park [33] investigated overtime hours in diverse medical departments in South Korea, revealing that nurses working daytime shifts in internal medicine wards worked more overtime than did those in specialized units. Weekly overtime was the highest in ear-nose-and-throat wards and the lowest in psychiatric wards. Similarly, in our study, the number of nurses working more than 50 h/week was lower in the intensive care unit than in internal medicine or surgery wards, presumably because its ratio of patients to nurses is relatively smaller, and working overtime is usually not necessary. Bae and Champion [34] found that working hours significantly differed by working environment—a significantly higher number of nurse practitioners working in hospitals and long-term care settings worked for more than 40 h/week. Long working hours are known to have detrimental effects on workers’ health and outcomes. Based on our findings, it is imperative that hospital nurse managers and health professionals perceive the impact of long working hours. Furthermore, if longer working hours are necessary because of the nature of the department or unit, it is desirable to adjust them to appropriate levels through various approaches, such as hiring more personnel or altering the nursing delivery system.

In the present study, the scores for PS competencies decreased with increasing working hours. This result is in line with previous findings that confidence in or perceptions about PS decline as working hours increase [32]. There is substantial evidence to suggest the possibility of the association of fatigue and shorter sleep with long working hours, which can lead to loss of concentration and perceived quality of care and PS [13]. A recent study on the effect of hospital nurses’ shift lengths on the perceived quality and PS [35] reported that longer working hours were negatively associated with care quality and safety. More prospective studies are needed to identify the mechanism linking long working hours and perceived or actual PS activities. Furthermore, the perceptions of adverse nurse outcomes by nurses working more than 50 h were significantly higher than those of nurses working less than 40 h. This suggests that working long hours exposes nurses to adverse outcomes, potentially having detrimental effects on PS. Olds and Clarke [11] conducted a similar study and found that all variables of adverse nurse outcomes were significantly correlated with errors in the group of nurses who worked more than 40 h/week. Moreover, medication error and needle stick injuries were associated with voluntary overtime and working hours. Based on these findings, they called for regulations specifying mandatory working hours. Direct comparisons between this study and previous research must be attempted with caution because of differing circumstances and contexts across countries. Nevertheless, it is of great significance that the results of this study can be used as a basis for establishing overtime rules in South Korea.

Having filed more than three PS accident reports in the past year, working more than 40 h/week, and having high PS competencies were significantly associated with adverse nurse outcomes. Of these, working more than 40 h had the most significant association. Long working hours are the most prominent issue in hospital organizations because they affect the safety of both patients and healthcare personnel [11]. Nevertheless, South Korea lacks regulations concerning limiting nurses’ overtime work. Some studies have suggested that long working hours undermine intelligibility while increasing fatigue [36,37]. Further, working overtime has been reported to induce fatigue and stress and facilitate burnout among nurses, while mandatory overtime was found to be related to lower organizational commitment [38]. In a study analyzing the relevance of depression to the working hours of public sector workers in South Korea [39], those working more than 10 h/day reported 1.63 times higher levels of depression than those working fewer than 10 h. In addition, they reported 2.2 times higher levels of depression when working nights. As such, it will also be necessary to confirm the relevance of overtime and mental health problems through further research. Hospital administrators must formulate policies to reduce overtime based on its link with mental health problems. Hence, our findings provide grounds for nurse leaders and policymakers to develop regulations on appropriate nurse staffing and mandatory overtime. Mostly, South Korean nurses employed at medium to large hospitals work in three daily shifts—eight hours each in the day, evening, and night. Thus, implementing various work schedules, such as night or day-only shifts and 12 h shifts, could be useful. In addition, various in-hospital efforts are needed to establish protocols regarding transition time in order to reduce overtime.

PS competency was another factor associated with adverse nurse outcomes. According to Alves and Guirardello [40], establishing a favorable working environment is a principal factor in organizational leaders’ and nursing departments’ roles in terms of PS and professional practices, which is in line with our findings. Hwang [23] also found that nurses with higher PS competencies perceived the PS atmosphere more positively, and emphasized that efforts that stress teamwork, which enhances nurses’ PS competencies, should be implemented. A recent study on self-reported PS competencies among new nursing graduates reported that greater confidence in PS implementation learning was associated with learning experiences in clinical settings [41]. Therefore, the development and implementation of intervention programs that cultivate and strengthen nurses’ PS competencies in hospital settings is essential to reduce adverse nurse outcomes. Furthermore, hospital administrators should strive to foster a non-punitive culture against the adverse events until settlements of healthy work environments including PS culture. 

The final factor that was found to be associated with adverse nurse outcomes was the number of accident reports in the past year. Nurses who had filed more reports were more frequently exposed to adverse outcomes, and this phenomenon can be understood in the context of the South Korean nursing organizational culture. In South Korean hospitals, reporting more than three PS events might stigmatize a nurse, and nurses may thus fear being punished. Nurses who file several accident reports may be subject to adverse treatment by the head nurse, which may undermine their focus toward nursing work, thereby inducing more adverse nurse outcomes.

The findings of this study have limited generalizability owing to a relatively small sample size with all female participants. In addition, all three hospitals from which data were collected are affiliated to the same university. Thus, further studies with wider inclusion criteria are needed to investigate all units in different-sized (small, medium, and large) and/or representative hospitals. Furthermore, because we did not objectively measure actual adverse nurse outcomes but only relied on nurses’ self-reported responses, the results may be considerably overestimated or underestimated. Therefore, there is a need for empirical studies utilizing objective indices for nurse outcomes or actual medical accident data. Furthermore, studies should investigate the potential associations of working hours and overtime with patient outcomes as well as whether PS competencies play a mediating role in the relationship between nurses’ working hours and adverse nurse outcomes. Lastly, in this study, weekly working hours were classified according to the KWCS criteria. However, such a categorization may not be ideal for determining optimal cutoff values for working hours to establish positive work environments. Accordingly, future studies must conduct receiver operating characteristic curve analysis to identify optimal cutoffs for working hours.

## 5. Conclusion

Our study highlights that nurses working fewer than 40 h/week had higher PS competencies than did those working more than 40 h/week. Further, nurses working more than 50 h/week showed the highest adverse nurse outcome scores. Our analysis revealed that having filed more than three PS accident reports in the past year, working more than 40 h/week, and having higher PS competencies were detrimental to nurse outcomes. In light of our findings, there is a need for more specific policies and measures to protect nurses and patients from the adverse outcomes associated with working overtime in hospitals. In addition, nurses themselves should try to participate in various activities geared toward the improvement of a PS culture and unit-level work environments. Furthermore, other intervening factors such as nurses’ burnout, peer relationships, and social support from nurse managers, patients, and families need to be examined. 

## Figures and Tables

**Table 1:** Participants’ characteristics by working hour group (*N* = 364).

Characteristics	Working Hours			χ^2^	*p*
	<40 (*n* = 54)	40–49 (*n* = 190)	≥50 (*n* = 120)		
	*n* (%)	*n* (%)	*n* (%)		
Age (years)					
<29	41 (11.7)	107 (54.1)	64 (34.2)	6.957	0.138
30–39	9 (8.1)	55 (49.6)	47 (42.3)		
≥40	4 (9.8)	28 (68.3)	9 (21.9)		
Marital status					
Married	41 (17.1)	114 (47.5)	85 (35.4)	4.976	0.083
Not married	13 (10.5)	76 (61.3)	35 (28.2)		
Education					
Diploma	15 (11.7)	67 (52.4)	46 (35.9)	1.319	0.858
Bachelor’s	33 (16.3)	101 (50.0)	68 (33.7)		
Master’s	6 (17.6)	22 (64.8)	6 (17.6)		
Units					
Medical ward	16 (14.3)	61 (54.5)	35 (31.2)	23.652	0.003
Surgical ward	3 (2.3)	64 (50.1)	61 (47.6)		
Intensive care unit	31 (34.8)	44 (49.5)	14 (15.7)		
Emergency room	3 (11.6)	16 (61.5)	7 (26.9)		
Hemodialysis room	1 (11.1)	5 (55.6)	3 (33.3)		
Position					
Staff	49 (14.5)	175 (51.8)	114 (33.7)	3.438	0.179
Management	5 (19.2)	15(57.7)	6 (23.1)		
Career length (years)					
1–5	21 (15.1)	73 (52.5)	45 (32.4)	9.994	0.125
5–9	25 (21.7)	55 (47.8)	35 (30.5)		
10–14	3 (5.9)	22 (43.2)	26 (50.9)		
≥15	5 (8.5)	40 (67.7)	14 (23.8)		
Experience of patient safety education					
Yes	49 (13.9)	183 (53.2)	114 (32.9)	0.483	0.785
No	5 (27.8)	7 (38.9)	6 (33.3)		
Instances of reporting patient safety accidents (within the last year)					
None	11 (12.2)	48 (53.3)	31 (34.5)	6.084	0.193
1–2	39 (20.5)	87 (45.8)	64 (33.7)		
≥3	4 (4.7)	55 (65.5)	25 (29.8)		

**Table 2:** Differences in patient safety competencies and adverse nurse outcomes by working hour group (*N* = 364).

Variables	Working Hours			F (p)
	<40 (*n* = 54)	40–49 (*n* = 190)	≥50 (*n* = 120)	
	Mean ± SD	Mean ± SD	Mean ± SD	
Patient safety competencies	56.00 ± 6.92	55.69 ± 7.60	53.31 ± 7.13	4.217 (0.015)
Adverse nurse outcomes	44.29 ± 7.07	46.35 ± 5.82	48.53 ± 5.58	7.163 (0.001)

SD, standard deviation.

**Table 3:** Factors associated with adverse nurse outcomes (*N* = 364).

Factors	Step 1				Step 2			
	*β*	*T*	*p*	95% CI	*β*	*t*	*p*	95% CI
Age (years) (reference ≥40)	0.033	0.386	0.700	−3.083 to 4.587	0.060	0.702	0.438	−2.240 to 5.147
Marital status (reference = not married)	0.016	0.247	0.805	−1.399 to 1.907	0.022	0.340	0.734	−1.304 to 1.849
Education (reference ≥ master’s)	−0.067	−1.164	0.245	−5.365 to 1.377	−0.059	−1.027	0.305	−5.059 to 1.589
Unit (reference = hemodialysis room)	−0.073	−1.361	0.174	−6.238 to 1.137	0.068	−1.278	0.202	−5.994 to 1.273
Position (reference = charge nurse)	−0.038	−0.597	0.551	−4.627 to 2.473	−0.035	−0.557	0.578	−4.487 to 2.507
Career length (years) (reference ≥15)	0.060	0.628	0.530	−1.672 to 3.242	0.062	0.665	0.506	−1.602 to 3.239
Experience of patient safety education (1 = no)	0.080	1.523	0.129	−.667 to 5.243	0.066	1.267	0.206	−1.040 to 4.800
Instances of reporting patient safety accidents (within the last year) (reference ≥3)	0.149	2.386	0.018	0.365 to 3.789	0.146	2.378	0.018	0.352 to 3.725
Working hours (reference ≥40)	0.223	4.188	<0.001	0.109 to 0.301	0.202	3.815	<0.001	0.090 to 0.281
Patient safety competencies					−0.179	−3.341	0.001	−0.225 to −0.058
Adjusted R^2^, *F (p),* △ R^2^	0.082, 3.03 (< 0.001), 0.122				0.109, 3.63 (< 0.001), 0.029			

CI, confidence interval.
